# Supplementary material for: DANCE provides an open-source and low-cost approach to quantify aggression and courtship in Drosophila
Source: eLife. 2025 Dec 29;14:RP105465. doi: 10.7554/eLife.105465 (PMC12747525; doi:10.7554/eLife.105465)
Supplement: MDAR checklist [file elife-105465-mdarchecklist1.pdf]

Westbrook Centre
Milton Road
Cambridge CB4 1YG, UK
P 01223 855340
W elifesciences.org
T @elife

Materials Design Analysis Reporting (MDAR) Checklist for Authors

Materials:

**Newly created materials**	**Indicate where provided: section/figure legend**	**N/A**
The manuscript includes a dedicated "materials availability statement" providing transparent disclosure about availability of newly created materials including details on how materials can be accessed and describing any restrictions on access.	Patent application entitled “Device for Measuring Complex Social Behaviors In Small Insects” based on this research was filed by the Manipal Academy of Higher Education to the Indian Patent Office (Application No. 202441072884).<br><br>We have described the design and making of the DANCE hardware setup for both aggression and courtship behaviors in the Methods section under the title “DANCE hardware”. Rest of the instructions on using the DANCE analysis pipeline are mentioned in the Methods section as well, and developed codes are publicly available on GitHub (https://github.com/agrawallab/DANCE).	

**Antibodies**	**Indicate where provided: section/figure legend**	**N/A**
For commercial reagents, provide supplier name, catalogue number and RRID, if available.		N/A

**DNA and RNA sequences**	**Indicate where provided: section/figure legend**	**N/A**
Short novel DNA or RNA including primers, probes: Sequences should be included or deposited in a public repository.		N/A

**Cell materials**	**Indicate where provided: section/figure legend**	**N/A**
Cell lines: Provide species information, strain. Provide accession number in repository OR supplier name, catalog number, clone number, OR RRID.		N/A
Primary cultures: Provide species, strain, sex of origin, genetic modification status.		N/A

**Experimental animals**	**Indicate where provided: section/figure legend**	**N/A**
Laboratory animals or Model organisms: Provide species, strain, sex, age, genetic modification status. Provide accession number in repository OR supplier name, catalog number, clone number, OR RRID.	**Species:** *Drosophila melanogaster*<br><br>Wild-type Canton S obtained from Ulrike Heberlein, HHMI, Janelia Research Campus. TH-GAL4, dilp2-GAL4, Dsk-RNAi, attP2 empty vector control from Bloomington Drosophila Stock Center, and UAS-GtACR1 obtained from Gaurav Das, NCCS, Pune.<br><br>We used both male and female fruit flies for rearing at standard conditions and 6 days old male flies for experiment.	
Animal observed in or captured from the field: Provide species, sex, and age where possible.		N/A

**Plants and microbes**	**Indicate where provided: section/figure legend**	**N/A**
Plants: provide species and strain, ecotype and cultivar where relevant, unique accession number if available, and source (including location for collected wild specimens).		N/A
Microbes: provide species and strain, unique accession number if available, and source.		N/A

**Human research participants**	**Indicate where provided: section/figure legend or state if these demographics were not collected**	**N/A**
If collected and within the bounds of privacy constraints report on age, sex, gender and ethnicity for all study participants.		N/A

Design:

**Study protocol**	**Indicate where provided: section/figure legend**	**N/A**
If the study protocol has been pre-registered, provide DOI. For clinical trials, provide the trial registration number OR cite DOI.		N/A

**Laboratory protocol**	**Indicate where provided: section/figure legend**	**N/A**
Provide DOI OR other citation details if detailed step-by-step protocols are available.		N/A

Experimental study design (statistics details) *		
**For in vivo studies: State whether and how the following have been done**	**Indicate where provided: section/figure legend. If it could have been done, but was not, write "not done"**	**N/A**
Sample size determination	Formal statistical sample size calculation is not typically applied in Drosophila studies and was not done. Sample sizes were based on standard practice in the field.	N/A
Randomisation	Randomisation was not performed because flies were assigned to groups based on predefined genotype and housing conditions.	N/A
Blinding	Blinding was not performed because genotypes were handled as separate cohorts.	N/A
Inclusion/exclusion criteria	Inclusion criteria were standard for Drosophila experiments (e.g., age-matched adult flies, correct genotype). Flies with physical deformities or handling injury were excluded.	

Sample definition and in-laboratory replication	Indicate where provided: section/figure legend	N/A
State number of times the experiment was replicated in the laboratory.	Experiments were replicated at least 2–3 times (2–3 independent biological replicates conducted on different days). Sample sizes are shown in Figure legends, and represent technical replicates (individual animals).	
Define whether data describe technical or biological replicates.	Data describe technical replicates, with "n" indicating the number of individual flies.	

Ethics	Indicate where provided: section/submission form	N/A
Studies involving human participants: State details of authority granting ethics approval (IRB or equivalent committee(s), provide reference number for approval.		N/A
Studies involving experimental animals: State details of authority granting ethics approval (IRB or equivalent committee(s), provide reference number for approval.	Experiments involving *Drosophila melanogaster* do not require approval from an IRB or equivalent committee(s); therefore, no ethics approval was sought or required.	N/A
Studies involving specimen and field samples: State if relevant permits obtained, provide details of authority approving study; if none were required, explain why.		N/A

Dual Use Research of Concern (DURC)	Indicate where provided: section/submission form	N/A
If study is subject to dual use research of concern regulations, state the authority granting approval and reference number for the regulatory approval.		N/A

Analysis:

Attrition	Indicate where provided: section/figure legend	N/A
Describe whether exclusion criteria were pre-established. Report if sample or data points were omitted from analysis. If yes, report if this was due to attrition or intentional exclusion and provide justification.	Attrition was not observed; exclusions were predefined and described in Methods.	

Statistics	Indicate where provided: section/figure legend	N/A
Describe statistical tests used and justify choice of tests.	See the Methods section entitled "Statistical analysis" and figure legends.	

Data availability	Indicate where provided: section/submission form	N/A
For newly created and reused datasets, the manuscript includes a data availability statement that provides details for access (or notes restrictions on access).	All data associated with this study are present in the paper, Supplementary Materials and Github repository: https://github.com/agrawallab/DANCE.	
When newly created datasets are publicly available, provide accession number in repository OR DOI and licensing details where available.		N/A
If reused data is publicly available provide accession number in repository OR DOI, OR URL, OR citation.		N/A
**Code availability**	**Indicate where provided: section/figure legend**	**N/A**
For any computer code/software/mathematical algorithms essential for replicating the main findings of the study, whether newly generated or re-used, the manuscript includes a data availability statement that provides details for access or notes restrictions.	Code and software generated in this study are publicly available at: https://github.com/agrawallab/DANCE	
Where newly generated code is publicly available, provide accession number in repository, OR DOI OR URL and licensing details where available. State any restrictions on code availability or accessibility.	https://github.com/agrawallab/DANCE	
If reused code is publicly available provide accession number in repository OR DOI OR URL, OR citation.	Re-used software used in this study are publicly available at: https://github.com/kristinbranson/JAABA https://github.com/kristinbranson/FlyTracker https://github.com/Dicksonlab/MateBook https://www.vision.caltech.edu/cadabra/	N/A

Reporting:

The MDAR framework recommends adoption of discipline-specific guidelines, established and endorsed through community initiatives.

**Adherence to community standards**	**Indicate where provided: section/figure legend**	**N/A**
State if relevant guidelines (e.g., ICMJE, MIBBI, ARRIVE, STRANGE) have been followed, and whether a checklist (e.g., CONSORT, PRISMA, ARRIVE) is provided with the manuscript.		N/A
